# “Optimizing Learning in Integrated Curriculum”—Comparative Effectiveness of Online and Face-to-Face Formative Assessments: Mixed Methods Study

**DOI:** 10.2196/84935

**Published:** 2026-05-05

**Authors:** Amira Salem Ismail, Sarah Mohamed Hussein, Mohamed El-Shafey, Ahmed Farid Al-Neklawy, Ahd A Mansour, Shatha Ghazi Felemban, Shimaa Elaraby

**Affiliations:** 1 Medical Education Department, Fakeeh College for Medical Sciences, Fakeeh Care Group, Alhamara, Jeddah, 21461, Saudi Arabia, 966 558243429; 2 Medical Education Department, Faculty of Medicine, Suez Canal University, Round Road, Ismailia, Egypt; 3 Clinical Sciences Department, MBBS Program, Fakeeh College for Medical Sciences, Fakeeh Care Group, Jeddah, Saudi Arabia; 4 Department of Public Health, Community Medicine, Environmental Medicine and Occupational Medicine, Faculty of Medicine, Suez Canal University, Ismailia, Egypt; 5 Physiological Sciences Department, MBBS Program, Fakeeh College for Medical Sciences, Fakeeh Care Group, Jeddah, Saudi Arabia; 6 Department of Anatomy and Embryology, Faculty of Medicine, Mansoura University, Mansoura, Dakahlia, Egypt; 7 Department of Anatomy and Embryology, Faculty of Medicine, Ain Shams University, Cairo, Egypt; 8 Medical Laboratory Sciences Department, MLS Program, Fakeeh College for Medical Sciences, Fakeeh Care Group, Jeddah, Saudi Arabia

**Keywords:** formative assessment, feedback, online assessment, assessment, student engagement

## Abstract

**Background:**

Assessment is a critical component of teaching and learning and serves as the foundation for how learners demonstrate success in achieving learning objectives. Formative assessments (FAs) and timely feedback play a crucial role in integrated curricula, whereas basic and clinical sciences are taught in a coordinated manner. Feedback-based FA supports student learning, and teachers can determine learning gaps to monitor progress in learning. Based on existing evidence, limited literature compared the effect of online versus onsite FA on summative performance in a fully integrated curriculum.

**Objective:**

This study aimed to examine the effectiveness of online versus on-site FAs and feedback on summative assessment in the integrated medical curriculum.

**Methods:**

This study used an exploratory mixed methods approach to delving into students’ experiences with face-to-face versus online FA and feedback, and its effect on their summative performance in the integrated Bachelor of Medicine, Bachelor of Surgery program. This study was conducted at Fakeeh College for Medical Sciences in Jeddah, Saudi Arabia. A total of 143 consenting students were recruited into the study. The students in the study were distributed voluntarily into 2 groups regardless of age, sex, or academic performance. Group 1 (n=92) was assigned to receive online FAs and immediate online feedback throughout the module using the Speedwell system. However, Group 2 (n=51) was assigned to receive onsite FAs and face-to-face feedback throughout the module in the examination hall in the college. The quantitative part of the study involved analyzing student scores of summative assessments in 2 groups exposed to online and onsite FA and feedback. The qualitative part aimed to explore students’ perceptions of FA and feedback.

**Results:**

The passing rate in summative examinations (quiz, midmodule, and final) was higher in the onsite group (61.2%, 51%, and 62.7%, respectively) compared with the online group (53.3%, 48.3%, and 45.7%, respectively). However, the difference was statistically significant only in the quiz examination. Four key themes were identified from the qualitative analyses regarding participants’ different experiences of FA and feedback: the accessibility of the examination format facilitates flexibility in learning; FA is a means of recognizing learning opportunities; FAs help shift student attitudes toward learning; and the last theme is opportunities for discussion and personalized feedback.

**Conclusions:**

This research sheds light on the intricate interplay between assessment modalities and student learning outcomes by demonstrating that onsite FA followed by onsite feedback is more effective than online FA and feedback in fostering student engagement and promoting deep understanding and improving students’ performance in summative examinations. Thereafter, this study contributes to the ongoing discourse surrounding effective assessment practices in contemporary educational settings.

## Introduction

The integrated curriculum is now a cornerstone of modern medical education, bridging the gap between theoretical knowledge and clinical practice through integration and embracing relevance [1]. However, the effectiveness of the integrated curricula depends heavily on the assessment strategies used that support deep learning and enhance the knowledge application and skill development [2]. These assessment strategies are a cornerstone in medical education, serving not only as a tool to evaluate learners’ achievement of curricular outcomes but also as a driver of learning itself [3]. In addition, assessment can regulate learning with the delivery of feedback as a formative assessment (FA) or document achievement as a summative assessment [4].

The FA plays a pivotal role in evaluating learning progress and enhances it through continuous feedback mechanisms [5]. In medical curricula, FAs take the form of short tests with different types of questions that represent the content across different disciplines [6]. The aim of this type of assessment is the application of knowledge in clinically relevant contexts [7]. The FA is introduced to students to support learning through the knowledge gaps and guide the improvement at the level of individual students and the curriculum too [8]. FA is usually accompanied by formative feedback. Although these 2 terms are distinct, FA and formative feedback are complementary strategies [9]. The FA refers to the activities and different types of questions that provide evidence of learning, and the feedback refers to the information provided in response to that evidence [10]. FA is widely used as a powerful tool that provides learners with prompt feedback that identifies strengths and learning gaps across different disciplines, which helps students to bring relevance to their study and makes it more authentic [11]. The ongoing feedback loop provided through FA promotes critical thinking, helps students to have self-regulation strategies, and ensures that students are better prepared for high-stakes summative assessments [12]. Importantly, FA in an integrated curriculum does not merely test discrete knowledge areas; rather, it encourages students to synthesize information and apply it in clinically relevant contexts. Moreover, the FA can help in identifying areas requiring further integration and application [13]. Research studies provide evidence that FA and feedback serve as valuable strategies that facilitate student understanding, promote enhanced cognitive engagement, and foster self-regulated learning [14-16]. However, these effects vary depending on the implementation strategies of FA and feedback [17].

As digital technologies have become more common in medical education, FAs and feedback are now given in both traditional face-to-face settings and online settings [18]. Online assessments offer flexibility, immediate feedback, and opportunities for adaptive learning, while face-to-face assessments foster interactive dialogue, personalized clarification, and the development of professional communication skills [19]. Despite these differences, evidence remains limited and inconsistent regarding the comparative effectiveness of these modalities in influencing students’ summative performance within an integrated curriculum [20].

Available evidence indicates that a positive and significant correlation exists between students’ scores in online formative tests and their performance in summative assessments. Notably, this relationship appears more pronounced among students with higher entry qualifications [21]. According to a previous study, individualized or personalized feedback received online was found to be more effective for several students compared with face-to-face feedback or peer feedback [22]. Although online feedback was found accessible and acceptable by the majority of the students, it is not enough to ensure student engagement and depends heavily on students’ self-regulation and motivation [23]. Nevertheless, limited research has been conducted comparing online FAs with automated feedback to onsite FAs with face-to-face feedback in the context of medical student performance in summative assessments [24]. Therefore, this study aimed at examining the effectiveness of online versus on-site FAs and feedback on summative assessment performance in a fully integrated curriculum. Moreover, the study explored the students’ perception and experience of both assessment and feedback modalities for a better understanding of the variation in the summative performance. The study hypothesis was that onsite FA followed by face-to-face feedback is associated with higher summative assessment performance than online FA and online feedback. The study hypothesis is supported by the socio-constructivist perspective that highlights the idea that knowledge develops through interaction, dialogue, and the shared construction of meaning. These processes tend to emerge more naturally in face-to-face settings, where learners and educators can engage in real-time discussion and collaborative sense-making. Additionally, onsite feedback enhances the affective domain of learning, including confidence, motivation, and instructor scaffolding [25]. Additionally, the explanatory sequential mixed methods design was implemented to test the hypothesis through a quantitative approach, followed by deep exploration through qualitative inquiries on how students perceived different modalities of FA and feedback.

## Method

### Study Approach

This study used an explanatory sequential mixed methods quasi-experimental design to examine the association between the format of FA and feedback (online vs onsite) and subsequent summative students’ performance. The study further sought to explore the students’ experience of onsite versus online FA and feedback on their summative performance. In accordance with the explanatory sequential mixed approach, the study was conducted into 2 phases. The quantitative phase was implemented to compare the summative assessment performance between students exposed to online versus onsite FA and feedback. The findings from the first phase informed the development of the qualitative component in 2 key approaches: first, the refinement of the interview guide to explore the differences in students’ experience and perceived feedback effectiveness of the 2 different modalities of FA and feedback. Additionally, the quantitative performance outcomes supported the purposive sampling to ensure the representation of students with different achievement levels. This design helps in gaining a more complete picture than a standalone quantitative or qualitative study, as it integrates the benefits of both methods. It facilitates adding qualitative questions to the quantitative part to explore the students’ different perspectives toward the face-to-face versus online FA [26]. The quantitative arm of the study involved analyzing student marks for summative assessment between 2 groups exposed to online and onsite FA and feedback. The qualitative arm aimed to explore student perceptions on this matter and was guided by an interpretive phenomenological methodology [26]. This approach assumes that individuals construct their behavior and attitudes in conjunction with their interpretation of the world around them, considering their social and cultural context. Therefore, this approach is considered valuable in understanding the student experiences associated with diverse modes of FA and feedback as investigated in this study.

### Study Settings

This study was conducted at Fakeeh College for Medical Sciences (FCMS) in Jeddah, Saudi Arabia. The research took place during the academic year 2019‐2020, over 6 weeks of the integrated Endocrine and Reproductive module delivered to medical students.

### Study Design and Participants Sampling

An online recruitment form was distributed to all third-year medical students, and 143 consenting students were recruited into the study. Thereafter, the students in the study were distributed voluntarily into 2 groups regardless of age, sex, or academic performance.

**Figure 1.**
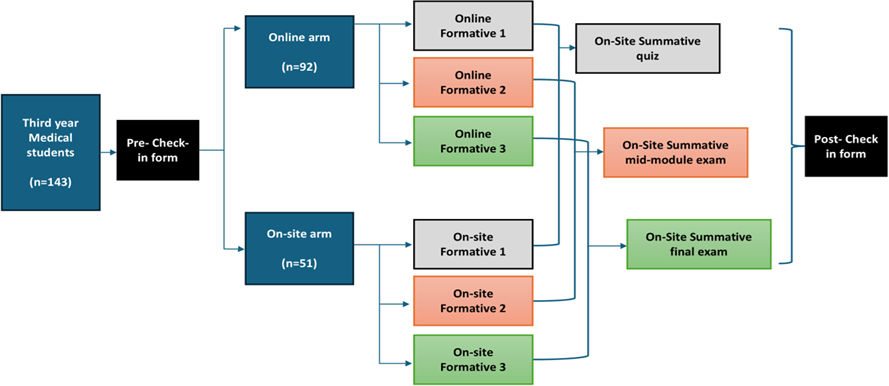
Distribution of the study groups of the undergraduate medical students at Fakeeh College for Medical Sciences, Jeddah, Saudi Arabia (2022), mixed method design (143 students).

Group 1 (n=92) was assigned to receive online FAs and immediate online feedback throughout the module using the Speedwell system, the online examination software used in the local educational setting (Speedwell Software Ltd., 2013‐2024). Speedwell System (Version 4.3.10088). Group 2 (n=51) was assigned to receive onsite FAs and face-to-face immediate feedback throughout the module in the examination hall in the college. All students completed the same summative assessments, as illustrated in Figure 1.

The FCMS uses a structured test blueprint for formative and summative examinations for the integrated Endocrine and Reproductive module. There was a total of 3 formative examinations taken throughout the 6 weeks of the module, each taken 3 days before the respective summative examination. The FA had the same number and types of questions as summative examinations. The FAs were conducted in weeks 2, 3, and 5, preceding the summative assessments, which included a quiz, midmodule examination, and final examination, respectively. The FAs consisted of a range of question types, including multiple-choice questions, modified essay questions, and long essay questions. The questions on the formative examinations were designed to assess learning and help provide feedback to students. The questions were based on what was learned during the module leading up to that point. The scoring of these questions varied based on assigned weights. The following table (Table 1) outlines the distribution of questions and scoring used in the FAs.

After the summative examination, 2 focus groups were conducted to explore participants’ experiences in relation to face-to-face FA and feedback as opposed to online modalities.

**Table 1.** The distribution of the number of questions and scoring used in the formative assessments (mixed method design).

Question types	Number	Score per question	Total score
MCQs^a^	10	1.5	15
MEQs^b^	1	2	2
LEQs^c^	1	3	3
Total	12		20

a MCQ: Multiple-Choice Question.

b MEQ: Modified Essay Question.

c LEQ: Long Essay Question.

### Data Collection

#### Quantitative Data

A baseline assessment of student perceptions on FAs and feedback was sought using a check-in form on the first day of the semester for comparison with a similar evaluation at the end of the module.

Following each FA, students took 3 summative assessments throughout the module (quiz, midmodule examination, and final examination at the end of the module). Scores on the summative assessment were then recorded at the end of each summative examination.

As students began to complete the formative or summative examinations, the researchers moved on to grading, recording scores, and providing feedback to students. The researcher kept a journal throughout the module to record observations and field notes. The observations were anything that happened while students were taking formative and summative examinations. Some common observations made were common mistakes students made on the quizzes, the amount and type of feedback delivered to students, and any adjustments or changes in feedback to meet the needs of students.

#### Qualitative Data

Study participants were purposively invited for a focus group discussion to explore further their face-to-face and online modalities of FA and onsite and online feedback. The consenting individuals (n=20) were randomly allocated to 2 focus groups, each group consisting of 10 students, a mix of individuals who had experienced both modalities of FA in the study design. The qualitative sample size was guided by the information power principle, which states that the more relevant information the study participants hold in relation to the study aim and objectives, the fewer participants are required [26]. This principle is influenced by the specificity of the research aims and the depth of the dialogue obtained during the focus groups. In this study, participants were purposively selected from students at the same level who had experienced both online and face-to-face FAs within an integrated course. Because these participants had direct and relevant experience with the assessment modalities under investigation, the interviews generated rich and focused data aligned with the study objectives. Data collection continued until sufficient informational depth was achieved to address the research questions.

The focus groups were conducted using structured interview guides. The guide was pilot tested with 5 students who were not included in the final study sample to ensure relevance and clarity of the questions. Minor modifications are applied for the guide, including wording and sequencing of questions based on feedback obtained from the pilot testing. The interviews were conducted in English.

The focus group discussions were conducted smoothly with a nonhierarchical atmosphere. The data collectors played the role of facilitators. They asked open-ended questions and established a 2-way dialogue, avoiding dominating the discussion. The students had an equal opportunity of participation without fear of judgment or punishment. The focus group was audio-recorded and transcribed verbatim.

### Data Analysis

#### Quantitative Data Analysis

The summative grades of all students who were enrolled in both arms of the study, namely those who underwent either online FAs or attended onsite FAs before the summative assessment, were gathered and recorded within an Excel spreadsheet. The statistical package of the social science statistical program software version 22 (SPSS Inc) was used for analyzing quantitative data. Descriptive statistics were used to assess the overall and group-specific performance in the summative assessment, in the form of percentages, median, and range. Additionally, inferential statistics were used to evaluate whether there were statistically significant differences in the pass rate of the summative examination between the 2 groups.

The summative examination marks did not adhere to a normal distribution, as determined by the Shapiro-Wilk test. Therefore, the Mann-Whitney *U* test was used to evaluate any differences in student performance between the online and onsite FA groups. The summative examinations (quiz, midmodule theory, and final theory) have different maximum scores. Therefore, to ensure standardization of results and to display the pattern of the scores for the online and the onsite groups, we transformed these row scores into Z-scores. The pass mark is 50% in all summative examinations.

#### Qualitative Data Analysis

The focus group discussions were transcribed verbatim and analyzed manually following Braun and Clarke’s [26] approach to thematic analysis [27]. An inductive coding approach was used to generate themes from data without a predefined theoretical framework. Two researchers engaged in independent coding of the transcripts to achieve data familiarization. To ensure consistency and credibility of the analytic approach, any discrepancies between their codes were addressed through discussion and consensus, resulting in a final codebook used for the analysis. The qualitative data analysis followed 5 stages: immersing in the data through repeated reading of transcripts, coding, combining preliminary codes into categories and subthemes, and formulating overarching themes that conveyed the meaning of the dataset, thereby facilitating the write-up of the qualitative data [27]. Throughout the study, researchers maintained reflexivity by engaging in note-taking [14,15,28]. This served to recognize individual perceptions related to the study and to ensure that conclusions were grounded in the data derived from the study.

### Ethical Considerations

The research design applied in our study was approved by the Institutional Review Board at DSFH (approval number: 282/IRB/2022). Informed consent to participate was obtained from all the participants. In addition, the participants were volunteers, and no compensation was provided. All participants’ information was handled in accordance with strict privacy and confidentiality standards; identifying details have been omitted, and any potentially identifiable information or images have been included only with explicit written informed consent.

## Results

### Quantitative Results

This study was carried out on a cohort of 143 third-year students enrolled in the endocrine module at FCMS. Fifty-one students were enrolled in the onsite group who received onsite FA and feedback, while 92 students were enrolled in the group who received online FA and feedback. Four students were absent from the summative quiz examination, and 5 students were absent from the summative midmodule examination.

There were 3 summative examinations: a quiz, a midmodule examination, and a final theory examination. Each examination was evaluated using a different grading scale, with scores out of 5, 10, and 20, respectively.

**Figure 2.**
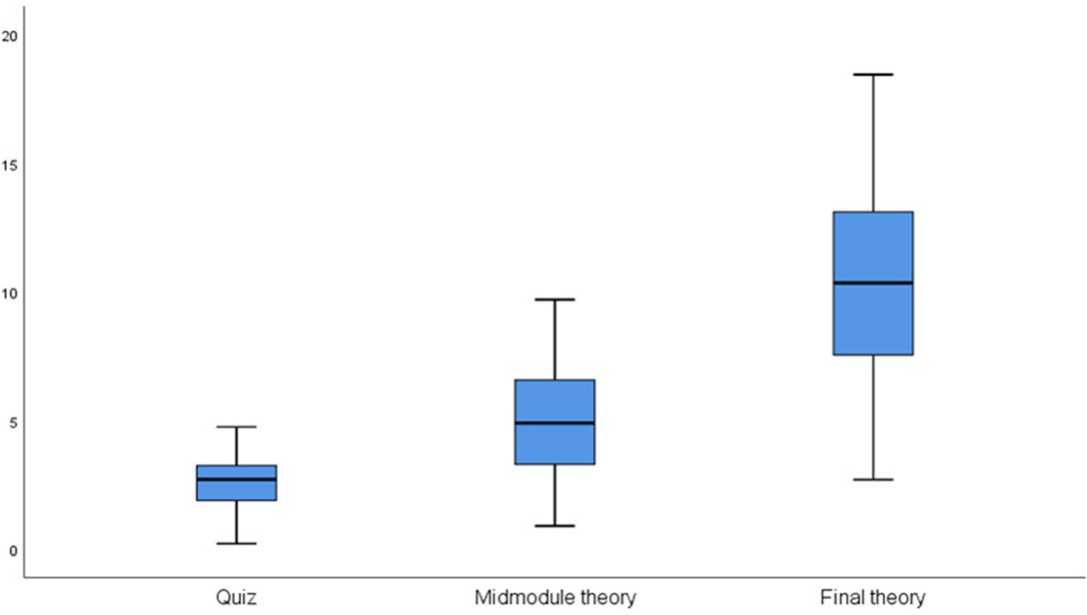
Distribution of student marks in 3 summative examinations assessed using different modes of delivery among undergraduate medical students at Fakeeh College for Medical Sciences, Jeddah, Saudi Arabia (2022), mixed method design (143 students).

The median and the range of marks obtained for each of the 3 summative examinations were calculated. The median of the quiz was 2.71, with a range from 0.21 to 4.75 marks. The median of the midmodule examination was 4.9, with a range from 0.90 to 9.70 marks. The final theory examination had a median of 10.35, and a range from 2.70 to 18.45 marks (Figure 2).

The median marks and range for the 3 summative examinations were calculated separately for the 2 student groups: the online FA and feedback, and the onsite FA and feedback groups. The onsite group had a higher median score of 2.80 marks (range 0.71 to 4.75) for the summative quiz compared with the online group, which received a median score of 2.64 marks (range 0.21 to 4.5). This difference was found to be statistically significant (*P*=.03).

There was no statistically significant difference in the median scores for the midmodule theory examination between the onsite group (4.95 marks, range 1.35 to 9.2) and the online group (4.90 marks, range 0.9 to 9.7).

**Figure 3.**
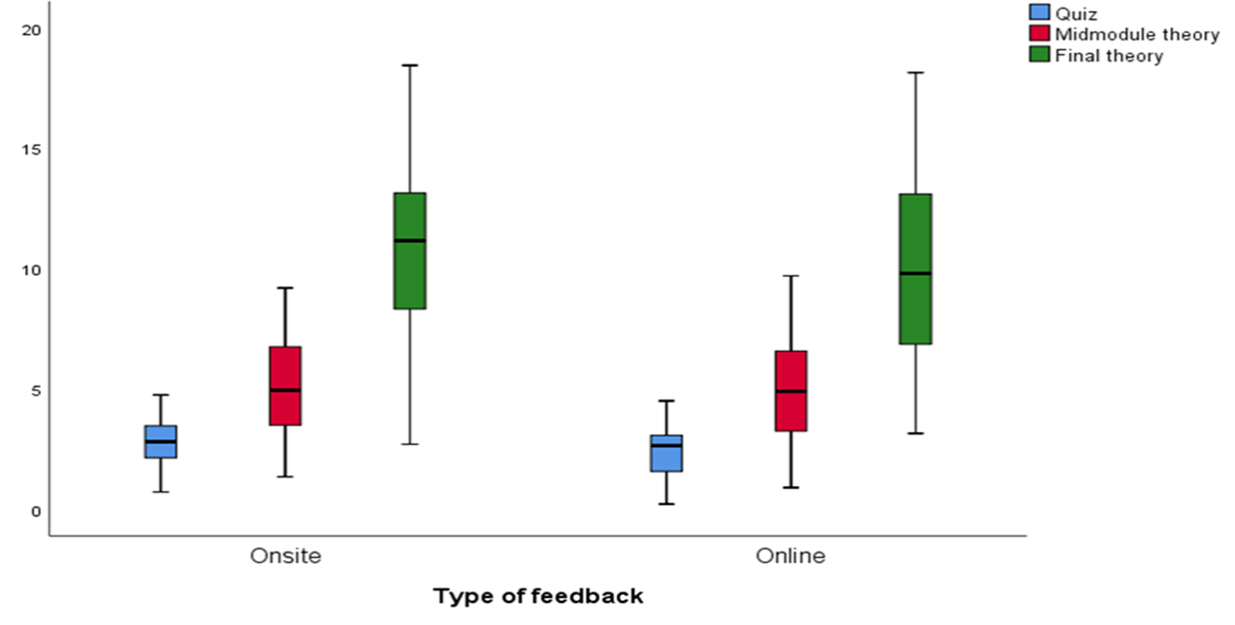
Distribution of student marks in the 3 summative examinations according to mode of formative feedback delivery among undergraduate medical students at a private medical college, Jeddah, Saudi Arabia (2022) (n=143 students).

The onsite group also had a higher median score for the final theory examination (11.16 marks, range 2.7 to 18.54) compared with the online group (median: 9.8 marks, range 3.15 to 18.50). However, this difference was not statistically significant (Figure 3).

The pass rates for the 3 summative assessments were calculated and compared between 2 cohorts of students: those who received online feedback and those who received onsite feedback. Table 2 illustrates the pass rates for each examination in both groups. The results indicate that the pass rate for the summative quiz was higher among students in the onsite group (61.2%) compared with those in the online group (53.3%). Similarly, the pass rate for the midmodule theory examination was higher in the onsite group (51%) compared with the online group (48.3%). However, these differences did not reach statistical significance. In contrast, the pass rate for the final examination theory was notably higher among students in the onsite group (62.7%) than among their online counterparts (45.7%). This discrepancy approached statistical significance, with a *P* value of .05 (Table 2). The pass mark is 50% in all summative examinations.

The summative examinations (quiz, midmodule theory, and final theory) have different denominators. Therefore, these scores were transformed into Z-scores to display the pattern of the scores for the online and the onsite groups and to conduct a standardized comparison.

**Table 2.** Pass rates of summative examinations according to mode of delivery among undergraduate medical students: a mixed method study at Fakeeh College for Medical Sciences, Jeddah, Saudi Arabia (2022) (n=143).

Summative examinations	Mode of delivery		*P* value^a^
	Onsite, n (%)	Online, n (%)	
Quiz examination (n=139): pass	30 (61.2)	48 (53.3)	.37
Midmodule theory examination (n=138): pass	25 (51.0)	43(44.3)	.76
Final examination theory examination (n=143): pass	32 (62.7)	42 (45.7)	.05

a Statistically significance was set at *P*<.05.

### Qualitative Results

The analysis of focus group discussions revealed 4 overarching themes that encapsulated participants’ diverse experiences of FA and feedback. These themes were derived from a total of 17 subthemes, offering further insights into the participants’ perspectives. These findings are presented below according to these 4 main themes in Table 3.

**Table 3.** Thematic analysis of the students’ focus groups mixed method study at Fakeeh College for Medical Sciences, Jeddah, Saudi Arabia (2022) (n=143).

Theme	Description	Students’ quotes
Theme 1: Accessibility of the examination format facilitates flexibility in learning.	This theme describes how the flexibility of examination timing and place contributed to its accessibility. Many participants expressed a preference for online formative assessments, citing their comfort and flexibility. Online assessments provide students with the convenience of completing their assessments at their own pace, allowing them to schedule their assessments around their academic workload and other commitments. This flexibility was not available with face-to-face assessments. Participants also noted that online assessments were less stressful since they eliminated the need for photocopying questions because they could revisit the examinations after submitting them. They also reported that online format was saving their time and provided more opportunities to reselect their answers. Therefore, there was a general consensus among the majority of participants that online formative assessments offered improved flexibility, accessibility, and reduced examination-related stress compared with traditional face-to-face assessments.	”I am totally with online formative assessment, online formative assessment more flexible and accessible for example, I will take the exam after I learned all the required material and the stress related to this is very low.” [FG1] ”I prefer online formative assessment, (it is) more comfortable to me and the questions were more accessible than the paper-based formative.” [FG1] ”The online formative assessment provides one with the opportunities to revisit the exam questions.” [FG1]
Theme 2: Formative assessment is a means of recognizing learning opportunities.	This theme describes the learning opportunities provided by the formative assessment and its effect on students’ preparation. Students acknowledged that formative assessment provided a valuable opportunity to assess their knowledge, identify any areas of weakness, and recognize the need for adjustments to their learning strategies. They found this process helpful in guiding them toward meeting the necessary standards and effectively preparing for future summative examinations.	“We test ourselves by taking the formative assessment and identifying our gaps in knowledge, so we can revisit some concept again before the summative.” [FG1] “The formative assessments guide me about the main concepts and topics that should be studied they also help me to improve my performance.” [FG1]
Theme 3: Formative assessments help shift student attitudes toward learning	The theme describes how formative assessment change students’ attitudes toward their study. The majority of students perceived online assessments as less stressful due to the flexibility it provided in scheduling examinations and the continued access to the assessment questions. This allowed for later reference and identification of individual learning needs, which was not possible in traditional face-to-face formative assessments. However, the reduced stress associated with online assessments was also found to lead to decreased commitment and motivation toward learning and studying for subsequent assessments. In contrast, onsite formative assessments simulated a similar stressful examination environment experienced during summative assessments. Although the associated stress in this context was seen as a drawback, it was considered beneficial in preparing students for the upcoming summative assessments. Overall, it was agreed upon by the majority that both online and onsite formative assessments influenced learning in positive and negative ways. However, it was believed that some level of examination-related stress was helpful in motivating students toward learning, making the onsite format more advantageous in this regard.	“I will take the exam after I learned all the required material, (therefore) the stress related to this is very low so, recalling of information was easy.” [FG1] “I feel online assessment gives me the opportunity to modify my learning style as availability of questions online makes me more comfortable in identifying my learning needs. However, it (online formatives) did not give me the real experience of the summative exam and most of the time I was not committed to study well before the formative assessment.” [FG1] “Being onsite and facing onsite formative assessment gives you the same experience of being in summative exam. With the stress related to exam… of course the stress related to the summative is much higher. Being in the same situation gives you the opportunity to test yourself in similar situations.” [FG2]
Theme 4: Opportunities for discussion and personalized feedback	This theme describes student perceptions toward feedback received through online and onsite modes. Students found the immediate provision of standardized feedback, including correct answers and the rationale was an advantage of online formative assessments compared with face-to-face feedback. However, this did not evoke the same level of enthusiasm among the majority of students. Face-to-face feedback allowed students to engage in discussions with their tutors, seek further clarification beyond the answers provided, and receive more personalized support. Hence, online feedback was considered less effective compared with face-to-face feedback due to the lack of communication and interactive discussion opportunities. Therefore, despite associated merits related to online feedback, the majority of students considered face-to-face feedback to be more beneficial.	“Feedback with the rationale of the right answer gives you opportunities about the good application exercises with discussion and rationale sometimes.” [FG2] “Online feedback was good however it lacks communication and discussions also elaboration. I think being committed and responsible for formative assessment affects your performance in summative assessment.” [FG1]

### Integration of Quantitative and Qualitative Results

A joint display was developed to demonstrate how qualitative themes provide contextual explanations for the quantitative results, as shown in Table 4.

The integration table provides a more comprehensive understanding of the role of FAs and feedback within the integrated curriculum. It indicates that although online FAs increase participation through flexibility and accessibility, onsite FAs may be more effective in supporting academic performance and examination preparedness due to opportunities for interaction, personalized feedback, and exposure to authentic assessment conditions.

**Table 4.** Joint display of integrated quantitative and qualitative results.

Quantitative results	Qualitative themes	Representative student perspectives	Integrated interpretation
The results demonstrated that 36% of the students were enrolled in the onsite group who received onsite formative assessment and feedback, while 64% of the students were enrolled in the group who received online formative assessment and feedback.	Accessibility of the examination format facilitates flexibility in learning	Students described that online formative assessment provide flexibility and convenience regarding timing and remote access	The students’ preference of online formative assessment is aligned with the student perspective that accessibility and convenience of online formative assessment increases flexibility in learning.
The results indicate that the pass rates for the 3 summative examinations were consistently higher among students in the onsite group. Moreover, in the quiz, the onsite group had higher median score than online group which was statistically significant.	Opportunities for discussion and personalized feedback	Students reported that onsite formative assessment and feedback allowed students to engage in discussions with their instructors, seek further clarification beyond the answers provided, and receive more personalized support. Hence, face-to-face feedback was considered more effective compared with online feedback due to the availability of communication and interactive discussion opportunities	Better performance and pass rates of face-to-face formative assessment aligns with the students’ perception that face-to-face formative assessment and feedback facilitate immediate clarification and interaction with instructors.
No statistically significant differences were observed in overall summative examinations scores between students who completed online versus onsite formative assessments except for the quiz.	Formative assessment is a means of recognizing learning opportunities	Students acknowledged that formative assessment provided a valuable opportunity to assess their knowledge, identify any areas of weakness, and recognize the need for adjustments to their learning strategies	Formative assessments support students in identifying learning gaps and preparing for summative assessments This leads to similar overall scores of summative examinations despite the mode of delivery.
Similar overall academic outcomes across the 2 modalities of formative assessment and feedback despite differences in pass rates.	Formative assessments help shift student attitudes toward learning	Students perceived online assessments as less stressful, which supported flexibility but sometimes reduced motivation. Conversely, onsite assessments created examination-like pressure that helped prepare them for summative examinations	While online assessments promote accessibility and reduce stress, onsite assessments may enhance motivation and examination preparedness by simulating the pressure of summative assessment environments. The qualitative data revealed, the formative assessment helps shift student attitude toward learning supporting the results that revealed similar overall academic outcomes across the 2 modalities.

## Discussion

### Principal Findings

This study used an explanatory sequential mixed methods quasi-experimental approach to investigate the differential impact of onsite versus online FAs and feedback on summative assessment performance. In this design, the qualitative results informed the qualitative inquiry that was used to explore and conceptualize the students’ experience and perception of the different formats of the FA and feedback. The effectiveness was assessed by comparing summative examination grades between students who received online and onsite formative feedback. Students in the onsite group achieved a higher passing rate across all summative assessments (quizzes, midterm, and final examinations) compared with their online counterparts. However, the differences were statistically significant only in the quiz examination.

Qualitative data provides important context for quantitative data interpretation, as the analysis of the data identified 4 recurring themes. Most of the students believed that online FA followed by online feedback was more flexible and accessible for them. The examination-related stress was reduced, especially with the opportunity for photocopying ideal questions and answers for revision. On the other hand, they reported that face-to-face FA followed by onsite feedback caused more stress because it was less accessible and not flexible. The participants also emphasized the importance of FA in identifying gaps that would help to improve their summative performance. Although both modalities facilitate learning, notable differences were observed in how students perceive the 2 experiences. Online assessments appear to promote self-directed learning and reduce anxiety. However, it was observed that onsite feedback was preferred by the study participants due to its ability to foster in-depth discussion and elaboration during solving application cases. This form of feedback motivated their learning to a greater extent compared with online feedback. These results also supported the quantitative data where superior summative quiz performance was observed in onsite FA and feedback, and these results reflect a better ability for students in this group to identify and address their learning gaps through more structured, engaged supervised experience, as the quiz was the first summative exposure for students in both groups. Additionally, combining the qualitative results with students’ perceptions and experience from the qualitative part conceptualizes the inconsistent outcome of online FA and feedback, although it was perceived as convenient and less stressful.

A multitude of research studies underline the significance of FA in enhancing subsequent student performance in summative assessments [14,15,28]. In addition to these findings, there is evidence of a positive association between students’ engagement in course activities and their final summative grades [24]. This evidence aligns well with the outcomes of this study, where participants consistently highlighted the value of FA and feedback in supporting their performance in summative assessments. Moreover, it has been confirmed that FA has a strong effect on the academic outcomes of poor learners [15]. However, the effectiveness of formative feedback is not clear-cut, as the effectiveness of feedback is likely to reflect variations with varying approaches and student characteristics [12]. This study sought to investigate this impact further by examining the impact of online and onsite FAs on students’ subsequent summative performance.

The literature on whether online feedback is superior to face-to-face feedback remains unclear, with existing studies frequently presenting contradictory findings. Previous studies indicate no significant differences in summative outcomes between learners who received online as opposed to onsite FAs [2,25,29]. However, other studies indicate a positive relationship between online formative test scores and summative assessment scores in students exposed to online formative test groups [30].

The students in this study expressed reduced stress while taking online FAs. This finding is supported by a study that examined the impact of online assessments on students’ behavior and beliefs in examinations. Additionally, according to Ice et al [31], students who received audio feedback had a higher percentage of satisfaction, relative to those who received their text feedback from online channels. The clarity of information conveyed through audio feedback, as well as instructors providing clearer direction, enthusiasm, and interactivity, were cited as facilitators of its perceived effectiveness [29].

However, in this study, the number of students who attended online FA and feedback was much higher than that of students who attended the onsite FA and feedback. Previous studies have indicated that the choice of adopting an online mode of assessment could facilitate students in self-regulation of learning, in contrast to the conventional onsite mode of delivery. This could be due to the access to instant feedback in the process of online FAs, which might ease the process of learning for learners and thus facilitate better academic performance by students on the following tasks [24,32].

In line with these findings, study participants acknowledged the benefits of online FAs and the subsequent feedback they received. However, the findings also revealed that the optional nature of online assessments was associated with a decrease in motivation toward learning. Consequently, student performance during the summative assessments varied. These findings suggest that online assessment and feedback may be more effective for students who possess higher levels of capability, motivation, and self-regulation in their learning process [25,27]. An earlier study has supported this claim and showed that the engagement of students in the learning process can only be made possible through students’ willingness and desire to be involved in their own learning [33].

One of these challenges, as indicated by the participants in the study, is the limited opportunities for communication, discussion, and meaningful engagement with tutors in seeking clarification on the feedback they received and on related topics. These findings align with a previous study that examined learners’ perceptions of the deficiencies in online learning. The study emphasized that although learners may perceive online learning as more comfortable, the effectiveness of communication is diminished due to the absence of nonverbal cues in online feedback [34]. The notion of incorporating nonverbal cues in the delivery of online feedback is an interesting idea worth exploring further, especially in the context of the benefits associated with this. Furthermore, it is worth noting that traditional modes of providing feedback, which involve personalized and comprehensive face-to-face interaction, as well as the supportive social environment they create, have been acknowledged to motivate students to perform at a higher level [34]. There is also evidence that when teacher feedback is coupled with student engagement, this will mediate the effect between student achievement and feedback [35]. It seems that online feedback is a new challenge in replicating these benefits. However, this perspective is being challenged as research suggests that the effectiveness of the learning process is not solely determined by the mode of information delivery, but rather by the quality of interaction among peers and teachers, as well as the way the instructional content is presented [35]. Accordingly, student satisfaction is prompted mostly by active discussions and engagement among module participants. This suggests that online modes of instruction have the potential to offer opportunities for such engagement. Further studies should aim to investigate how online assessments can be proposed to provide experiences similar to those offered in traditional face-to-face settings, to enhance students’ overall learning.

Various studies have explored the challenges that tutors face when interacting with students online. These challenges have many aspects, including but not limited to the psychological aspects of the use of technology, the quality of faculty teaching relative to technical literacy, and the readiness of technical infrastructure. This is all established as an important requirement [36,37]. In addition, another challenge in online delivery is the requirement for effective pedagogical strategies to provide a variety of engagement opportunities, thus promoting motivation and creating a meaningful learning experience for students [38]. This suggests that online modality in teaching and learning can be challenging due to the feelings of the students as disconnected from their classmates and instructors if interactions are not provided. These findings emphasize the importance of continued faculty development and the necessity to explore the perceptions of teachers, the challenges they face, and potential strategies to bridge any gaps associated with the delivery of online teaching and assessments. However, these aspects were not specifically addressed in this study; they warrant consideration for future research [29].

### Limitations

Several factors may have influenced the study outcomes and should be considered when interpreting the findings. Student-related variables, such as motivation and self-regulation skills, were not formally measured and could act as potential confounders. Additionally, allowing students to optionally select the assessment and feedback format may have introduced variability in engagement. The quasi-experimental design also limits causal inference due to the absence of randomization and control over confounding variables. Moreover, the qualitative data did not specifically explore how FA and feedback affected preparation for summative assessments. The use of nonprobability, voluntary sampling further limits the generalizability of the results. Finally, although qualitative coding was conducted collaboratively with disagreements resolved through discussion and consensus, formal interrater reliability statistics were not calculated. While consensus-based coding is widely accepted in qualitative research, the lack of a formal reliability measure may reduce the reproducibility of the analytic process.

### Conclusions

The onsite formative examination improves the performance of students in summative examinations more than online FA. The qualitative results revealed that onsite FA and feedback are better for deeper understanding and long-term knowledge retention, especially in an integrated module, while the online format offers more flexibility, accessibility, and reduces anxiety. These results suggest revisiting the approach of using FA in teaching and learning. However, given the presence of unmeasured confounders, further research is needed to clarify the mechanism of improving the summative performance through a specific FA and feedback format.

## Abbreviation

FA: formative assessment
FCMS: Fakeeh College for Medical Sciences

## References

[1] Bader M, Burner T, Hoem Iversen S, Varga Z (2019). Student perspectives on formative feedback as part of writing portfolios. Assess Eval High Educ.

[2] Mitra NK, Barua A (2015). Effect of online formative assessment on summative performance in integrated musculoskeletal system module. BMC Med Educ.

[3] Black P, Wiliam D (2009). Developing the theory of formative assessment. Educ Asse Eval Acc.

[4] Black P, Wiliam D (1998). Assessment and classroom learning. Assess Educ Princ Policy Pract.

[5] Sadler DR (1989). Formative assessment and the design of instructional systems. Instr Sci.

[6] Goodwin RL, Nathaniel TI (2023). Effective feedback strategy for formative assessment in an integrated medical neuroscience course. Med Sci Educ.

[7] Brenner JM, Bird JB, Willey JM (2017). Formative assessment in an integrated curriculum: identifying at-risk students for poor performance on USMLE step 1 using NBME custom exam questions. Acad Med.

[8] Wander B, Fernandes ATF, Daudt CVG, Gomes MQ, Pinto MEB (2022). Curriculum integration in the formative assessment of distance continuing medical education: the use of integrative activities. Rev Bras Educ Med.

[9] Gikandi JW, Morrow D, Davis NE (2011). Online formative assessment in higher education: a review of the literature. Comput Educ.

[10] Xiao Y, Yang M (2019). Formative assessment and self-regulated learning: how formative assessment supports students’ self-regulation in English language learning. System.

[11] El-Yassin HD (2015). Integrated assessment in medical education. J Contemp Med Sci.

[12] Lipnevich AA, Mattern K, Feddock C (2025). Formative assessment and feedback in medical education: a practical guide: AMEE guide No. 189. Med Teach.

[13] Hattie J, Timperley H (2007). The power of feedback. Rev Educ Res.

[14] Shute VJ (2008). Focus on formative feedback. Rev Educ Res.

[15] Cohen A, Singh D (2020). Effective student feedback as a marker for student success. SAJHE.

[16] Bennett RE (2011). Formative assessment: a critical review. Assess Educ Princ Policy Pract.

[17] Brookhart SM (2007). Formative Classroom Assessment: Theory into Practice.

[18] Birenbaum M, DeLuca C, Earl L (2015). International trends in the implementation of assessment for learning: Implications for policy and practice. Policy Futures Educ.

[19] Li H (2016). How is formative assessment related to students’ reading achievement? findings from PISA 2009. Assess Educ Princ Policy Pract.

[20] Moreno J, Pineda AF (2020). A framework for automated formative assessment in mathematics courses. IEEE Access.

[21] Yan Z, Brown GTL (2021). Assessment for learning in the Hong Kong assessment reform: a case of policy borrowing. Stud Educ Eval.

[22] Robertson SN, Humphrey SM, Steele JP (2019). Using technology tools for formative assessments. JEO.

[23] Bhagat KK, Spector JM (2017). Formative assessment in complex problem-solving domains: the emerging role of assessment technologies. J Educ Techno Soc.

[24] Ozan C, Kincal RY (2018). The effects of formative assessment on academic achievement, attitudes toward the lesson, and self-regulation skills. Educ Sci Theory Pract.

[25] Creswell JW (2009). Qualitative, Quantitative, and Mixed Methods Approaches.

[26] Braun V, Clarke V (2013). Successful Qualitative Research: A Practical Guide for Beginners.

[27] Stodel EJ, Thompson TL, MacDonald CJ (2006). Learners’ perspectives on what is missing from online learning: interpretations through the community of inquiry framework. IRRODL.

[28] Azam F, Irshad K, Shaheen A, Moin H, Javed N, Ahmer H (2021). Online versus conventional paper based formative assessment: do they predict summative scores?. Pak J Phsyiol.

[29] Cassady JC, Gridley BE (2005). The effects of online formative and summative assessment on test anxiety and performance. J Technol Learn Assess.

[30] Henly DC, Reid AE (2001). Use of the web to provide learning support for a large metabolism and nutrition class. Biochem Molecular Bio Educ.

[31] Ice P, Curtis R, Phillips P, Wells J (2007). Using asynchronous audio feedback to enhance teaching presence and students’ sense of community. J Asynchronous Learn Netw.

[32] Miller RL, Amsel E, Kowalewski BM, Beins BC, Keith KD, Peden BF (2011). Volume 1: Programs, Techniques and Opportunities.

[33] Yang L, Chiu MM, Yan Z (2021). The power of teacher feedback in affecting student learning and achievement: insights from students’ perspective. Educ Psychol (Lond).

[34] Al Kuhayli H, Pilotti M, El Alaoui K, Cavazos SE, Hassan SA, Al Ghazo R (2019). An exploratory non-experimental design of self-assessment practice. Int J Assess Eval.

[35] Mulvenon S (2021). A critical review of research on formative assessment: the limited scientific evidence of the impact of formative assessment in education. Pract Assess Res Eval.

[36] Zhao CG, Liao L (2021). Metacognitive strategy use in L2 writing assessment. System.

[37] Yan X, Zhang C, Fan JJ (2018). “Assessment knowledge is important, but …”: how contextual and experiential factors mediate assessment practice and training needs of language teachers. System.

[38] van der Vleuten CPM, Schuwirth LWT, Driessen EW (2012). A model for programmatic assessment fit for purpose. Med Teach.

